# Association of SP Educators (ASPE) Physical Examination Teaching Associate (PETA) Standards of Best Practice (SOBP)

**DOI:** 10.1186/s41077-025-00373-z

**Published:** 2026-02-19

**Authors:** Holly Hopkins, Tim Webster, Karen Lewis, Cathy M. Smith, Marsha E. Yelen

**Affiliations:** 1 Eastern Michigan University School of Nursing, 322 Marshall Building, Ypsilanti, MI 48197 USA; 2 Clinical Learning & Simulation Program, Rady Faculty of Health Sciences, University of Manitoba, 727 McDermot Avenue, Winnipeg, MB R3E 3P5 Canada; 3 University of Texas at Tyler School of Medicine, 11937 US Hwy. 271, Tyler, TX 75708 USA; 4 Baycrest Academy for Research and Education at Baycrest Centre for Geriatric Care, 3560 Bathurst Street, Toronto, ON M6A 2X8 Canada; 5 Rush University Medical Center, 1620 W. Harrison St, Chicago, IL 60612 USA

**Keywords:** Physical examination teaching associate, Standardized patient, Standardized patient methodology, Simulated patient/participant, Professional patient, Patient instructor, Patient educator, Patient partner, Physical examination instruction, Musculoskeletal examination

## Abstract

Physical Examination Teaching Associates (PETAs) instruct healthcare professional learners to perform accurate and respectful physical examinations. PETAs are trained to teach physical examination techniques to learners in a standardized manner while providing ongoing feedback to the learner based on the PETA’s experience receiving the examination with their own body. While PETAs fall under the broad umbrella of standardized/simulated patient/participant (SP) methodology, their distinct role necessitates specific Standards of Best Practice (SOBP) tailored to PETA programs. To address this need, the Association of SP Educators (ASPE) used the Delphi process, involving thirteen PETA methodology experts from four countries. The five Domains of the ASPE SOBP as well as the accompanying Principles and Practices were revised to align with the needs of PETA programs. There were modifications to terms and content to reflect the unique scope of PETA practice. The ASPE PETA SOBP is designed for programs where PETAs are involved in formative instructional sessions with learners. As with the prior ASPE SOBP, this aspirational document provides flexibility to adapt to diverse program contexts and will continue to evolve.

**Supplementary Information:**

The online version contains supplementary material available at 10.1186/s41077-025-00373-z.

## Introduction

Health professional learners obtain knowledge and skills related to physical examination techniques in many ways. One method of instruction is the application of physical examination teaching associate (PETA) methodology. PETAs are trained to teach physical examination techniques to learners in a standardized manner while providing ongoing feedback to the learner based on the PETA’s experience receiving the examination with their own body. PETA methodology, first reported in 1977, evolved alongside the growing application of standardized/simulated patient/participant (SP) methodology [1, 2]. In its infancy, SP methodology was also adapted to support instruction of other physical examinations, resulting in gynecological teaching associate (GTA) [2] and male urogenital teaching associate (MUTA) methodologies [1, 3, 4]. Although guidelines have been developed for SP [5] and GTA/MUTA [6] methodologies, this is the first time they are being articulated for PETA methodology.

SP methodology is a large umbrella encompassing the work of SPs, PETAs, GTAs, and MUTAs. As the global organization focused on SP methodology, the Association of SP Educators (ASPE) role is to identify and maintain evidence-informed standards for how to work with the SPs in their programs, including PETAs, GTAs, and MUTAs. ASPE published the Standards of Best Practice (SOBP) in 2017 [5] to provide evidence-informed guidance for supporting SP Educators in their work. Noting the distinction between engaging in role portrayal within a simulation and leading instructional sessions, the ASPE GTA/MUTA SOBP [6] were published to provide guidance for programs where GTAs/MUTAs instruct health professional learners to perform respectful and accurate breast, pelvic, urogenital, and rectal/prostate examinations. The ASPE PETA SOBP will provide similar support for programs where PETAs instruct health professional learners to perform other types of physical examinations (Table 1).

**Table 1:** Abbreviations and Prior SOBP Iterations

Phrase	Abbreviation	Relevant Publication
Association of SP Educators	ASPE	The ASPE SOBP was originally published in 2017 to support SP programs that engage in role play [5]. An updated version of the ASPE SOBP is forthcoming.
Standards of Best Practice	SOBP	
Gynecology Teaching Associate	GTA	The ASPE GTA/MUTA SOBP was published in 2021 to support GTA/MUTA programs where GTAs/MUTAs instruct learners to perform breast, pelvic, urogenital, rectal, and/or prostate examinations [6].
Male Urogenital Teaching Associate	MUTA	
Physical Examination Teaching Associate	PETA	The ASPE PETA SOBP is presented here to support PETA programs where PETAs instruct learners to perform physical examinations, such as musculoskeletal, head-to-toe, neurological, and/or cardiovascular.

A scoping review was conducted separately to review articles related to the implementation and utilization of PETA programs [7]. Full-text journal articles published in English before March 18, 2021, were included in the scoping review. Thirty-four articles were included (7). The findings of the scoping review focused on (a) how health professional learners engaged with PETA programs, (b) broad outcomes of PETA publications, and (c) whether the article reported on aspects of the Domains of the ASPE SOBP (5) and/or the ASPE GTA/MUTA SOBP (6). Some features will be summarized here, but full reporting is available in the published review [7].

PETA methodology was developed alongside and informed by SP methodology [2]. In 1977, Frazer and Miller published the first report of a PETA program [1]. Applying knowledge from SP and GTA methodology, they designed a program with an organ system approach and defined that PETAs would “serve as subjects on whom students would practice prior to meeting with physicians, instruct students in the techniques of performing physical examinations before their scheduled evaluation sessions with physicians, and evaluate students’ performances both from objective predetermined criteria and from their subjective points of view” (1, p. 149). Next, Laguna and Stillman (1978) reported on physicians training PETAs to instruct and evaluate medical students’ performance of the neurological examination [8]. Stillman went on to publish several additional articles applying PETA methodology to cardiovascular, musculoskeletal, orthopedic, pulmonary, neurologic, and complete physical examinations of individuals who were healthy and those with pathology [2, 9–13]. This seminal work remains the foundation and basis of PETA methodology.

PETAs have been documented as working in Australia [14], Canada [15], England [16], Germany [17], and the USA [18]. Publications demonstrate that terminology related to, and application of, PETA methodology varies widely [7, 19], but the foundations of the methodology as defined above remain consistent. After PETAs acquire the knowledge and skills to teach physical examination techniques, PETAs primarily work independently with small groups of medical students, nurse practitioner students, and physicians. PETAs may be healthy individuals or those with known pathology [13, 19, 20].

PETA program-level outcomes, learner perceptions, learner assessment post-instruction, and the experience of the PETA have been addressed, to varying degrees. Overall, outcomes were positive, but inconsistent reporting challenges the identification of program characteristics that best supported positive outcomes. Further research is needed.

Although PETA methodology evolved from SP methodology and many PETAs may work within an SP program, there are key differences in what they do. SPs are carefully trained to portray a role (e.g., patient), engage with learners while in this role, and sometimes provide feedback to and assessment of learners related to this interaction. On the other hand, PETAs instruct learners to perform physical examination techniques by providing ongoing feedback as the learner examines the PETA’s body [1, 7, 21]. PETA and GTA/MUTA methodology have more in common with each other. GTA/MUTA instruction addresses breast, pelvic, urogenital, rectal, and prostate examinations [6, 21], while PETA instruction addresses other physical examinations, such as musculoskeletal, head-to-toe, neurological, and/or cardiovascular. The PETA’s unique role indicates a critical need for evidence-informed standards to foster high-quality teaching and learning opportunities. Promoting physical and psychological safety is essential for all relevant parties (e.g., PETAs, learners, and future patients) and has effective outcomes aligned with institutional, governmental, and other regulatory policies.

**Fig. 1:**
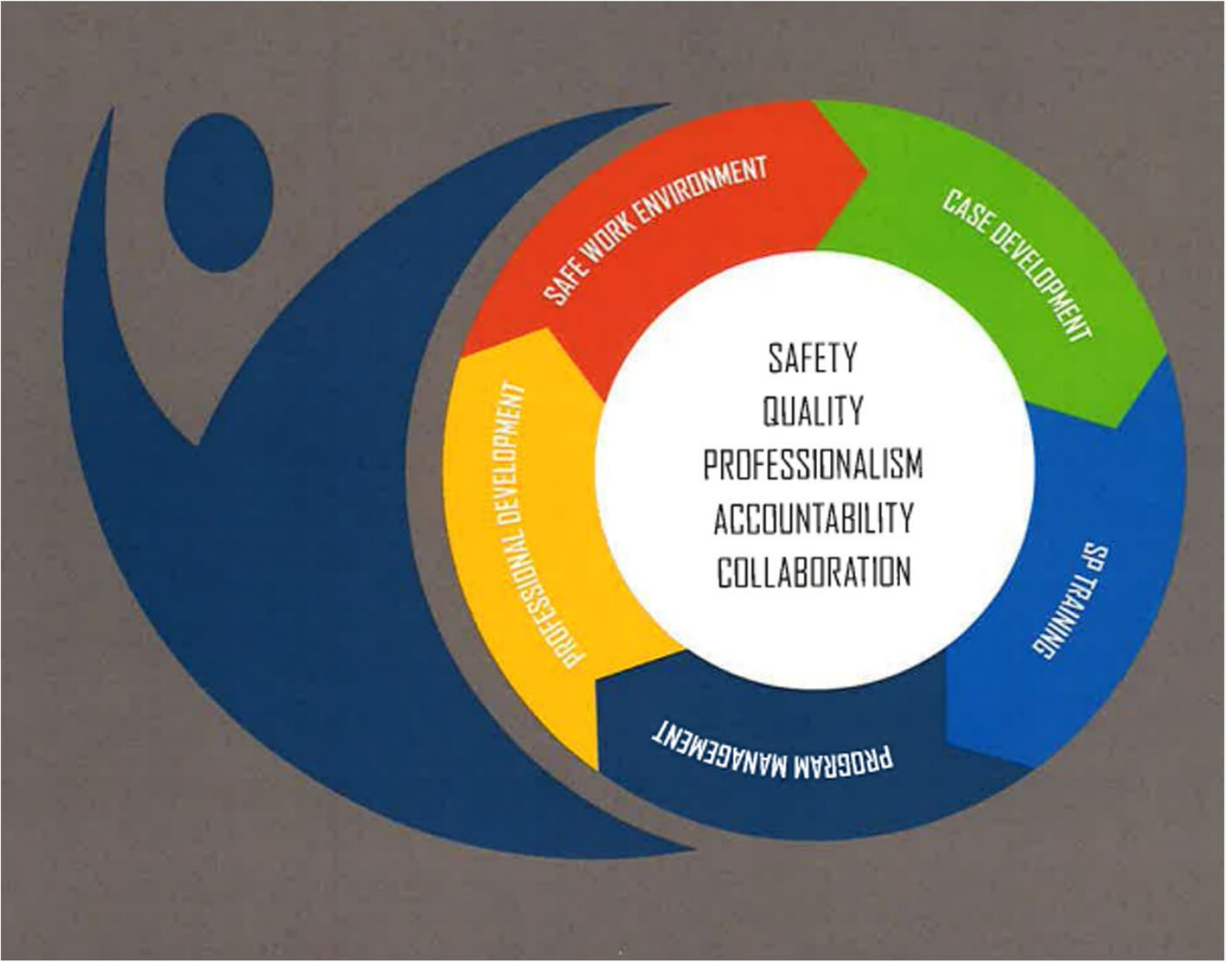
ASPE underlying values and domains

The aim of this paper was to publish evidence-informed SOBP pertaining to PETA programs to foster safe, high-quality teaching and learning opportunities. The ASPE PETA SOBP builds on the foundation of the ASPE SOBP [5] using the research methods employed to create the ASPE GTA/MUTA SOBP [6]. Because there are overlaps of the SP, PETA, GTA, and MUTA roles, there will be many similarities in this iteration of the SOBP, including language used; however, the differences within this document support the unique work occurring within PETA programs. As with the two other versions of the ASPE SOBP, in this document, there are five foundational values (safety, quality, professionalism, accountability, and collaboration) and five Domains [5] (Fig. 1). The Domains, along with accompanying Principles and Practices, have been modified in places to reflect the unique scope of PETA practice. For example, while Domain 2 in the ASPE SOBP addresses Case Development [5], Domain 2 in the ASPE PETA SOBP focuses on Instructional Session Development.

Similar to prior SOBP [5, 6], this document is an aspirational guide, and implementation will vary depending on each institution’s context. The SP Educator must identify and apply the appropriate SOBP(s) for their program needs. Where PETA programs exist within broader SP programs, the PETA program should align with the whole as much as is practical.

## Terms related to PETA methodology

*Instructional session*: a time when a PETA instructs learners about relevant physical examination content, safe examination techniques, and communication skills. A learner should receive at least minimal preparation (e.g., lecture, video) before an instructional session, and the PETA supports the development of learners’ knowledge, competence, and confidence through hands-on practice with immediate feedback [6, 22].

*Learner*: an individual (e.g., health professional, practicing health professional, PETA trainee) who will receive instruction from a PETA [6].

*Participant*: an individual actively engaged in an instructional session including the PETA and learner(s) [6].

*Physical Examination Teaching Associate (PETA)*: an individual (e.g., SP or community member) who is specifically trained to teach physical examination techniques in a standardized manner with their own body. Within a formative context, learners perform the physical examination techniques on the PETA. The PETA simultaneously provides real-time feedback to the learner based on their experience receiving the exam, which may be incorporated into subsequent attempts within the instructional session. They also teach learners the communication skills to conduct the exam with dignity and respect. PETAs work to cultivate a supportive, non-threatening learning environment. Some PETAs may have stable pathology present, but they teach more broadly about physical examination techniques beyond their own unique presentation [7].

*Physical Examination Teaching Associate (PETA) Program*: refers to any collection of PETAs working together with an SP Educator, whether within an SP program or as part of a stand-alone program.

*Relevant parties*: an individual or group (e.g., PETA, staff, faculty, client, learner, institution) impacted by PETA sessions.

*SP Educator*: “those who work to develop expertise in SP methodology and are responsible for training and/or administering SP-based simulation. Some may be trainers who exclusively work with SPs, while some may be faculty or healthcare professionals who work with SPs as part of their clinical and/or academic roles” [5]. Individuals overseeing and administering PETA instructional sessions are SP Educators. Alternative terms may include Human Simulation Expert [23].

## Methods

The ASPE Standards of Practice Committee used research methods previously established during the creation of the ASPE GTA/MUTA SOBP [6]. The authors of this article are committee members who were selected based on their interest in defining and promoting ASPE Standards of Practice. Authors are from Canada (CS, TW) and the USA (HH, KL, MY) and have expertise in PETA (KL), SP (KL, CS, TW, MY), and GTA/MUTA (HH, TW) methodology as well as experience developing the ASPE SOBP [5] (KL, CS) and the ASPE GTA/MUTA SOBP [6] (HH, TW).

## Ethics approval

The Eastern Michigan University Human Subjects Review Committee granted Exempt status, UHSRC-FY20-21–126.

### Delphi process

The Delphi Process was established to structure group communication and reach consensus on complex problems [24]. This approach typically involves an iterative series of surveys paired with anonymous, summarized participant feedback. Such structure engages participants regardless of location, allowing all voices to be heard. The primary limitation is the risk of participant attrition over time [25, 26]. The Delphi Process is increasingly utilized to identify consensus in medical education and medicine [27]. While there are many differences in how the Delphi Process is implemented [27, 28], the procedure established for the ASPE GTA/MUTA SOBP [6] was used here.

### Panelist recruitment

The Committee identified experts in PETA methodology through a variety of sources, including authors who have published on PETA methodology, ASPE members and contacts, and PETA programs with an online presence. ASPE, as an international organization, emphasizes the importance of participation from individuals of diverse locations and backgrounds. We directly invited 29 individuals from 7 countries to participate: Australia, Canada, Chile, Germany, Singapore, the UK, and the USA. Email invitations were also distributed via the ASPE List-serve. The invitation outlined the goals, the modified Delphi process, the anticipated timeline, and the inclusion criteria:

● >3 years of experience within a PETA program where PETAs instruct independently

●In one or more of the following roles:

○PETA (instructing learners)

○PETA educator/trainer (instructing PETAs)

○PETA program administrator

●Fluent in English (written)

●No conflict of interest

While the SOBP are intended to support SP Educators, PETAs were invited to represent their distinct and valuable perspectives. Individuals emailed the Committee to express interest and verify how they met the inclusion criteria. To promote diverse perspectives, only one representative was selected to participate from each PETA program. We selected a balance of experience among PETAs, PETA educators/trainers, and PETA administrators to represent multiple perspectives. Panelists remained confidential throughout the survey process, and feedback was summarized to reduce panelist identification. This method helps equalize all perspectives.

Because there is no standard number of participants to engage in the Delphi Process [27], researchers must identify the number that best fits their topic [29]. We aimed to have 15 panelists complete the Delphi Process, consistent with the ASPE GTA/MUTA SOBP [6].

### Survey completion

The ASPE SOBP [5] were used as the framework to establish the surveys. Panelists completed three iterative rounds of surveys within Survey Monkey, Inc. (2019) in 2021. The initial survey had two parts. The first part asked panelists to rate each ASPE SOBP Practice as it pertains to PETA programs on a 5-point scale (Not Important/Applicable, Minimally Important, Somewhat Important, Very Important, Critically Important); panelists were invited to provide written comments and rationale as desired. The second part of the survey asked panelists to brainstorm as many Practices as possible that are relevant to PETA methodology but not included in the original ASPE SOBP. Identical items were removed. Revisions to suggested items were made only for clarity while retaining the intended meaning. The proposed list was consolidated into the preexisting list of ASPE SOBP Practices and included within the second and third rounds of surveys.

Written comments from the prior survey round(s) were summarized and provided alongside the mean and standard deviation at the introduction to the second and third surveys (30). The feedback included causal rationale (e.g., reflection on the importance of physical autonomy) but not opinion-based rationale (e.g., an institution's unique policy) [30, 31].

Each survey was available for 2 weeks with email reminders sent 10 days after each survey was released. Only panelists who completed the prior survey round were invited to participate in the subsequent survey round.

### Defining consensus

The Committee used descriptive statistics in SPSS (version 26) to analyze the quantitative ratings. Consensus was defined as a mean of 3.8 on a 5-point scale with no participant rating an item as “Not Applicable” There is no evidence-based cut-off when identifying consensus using the Delphi Process; however, 75% is the median threshold used [28].

The Committee presented the proposed ASPE PETA SOBP at the ASPE 2021 Annual Conference to obtain feedback. All conference attendees were invited to participate in or watch a recording of a virtual presentation that included a description of survey results and the opportunity to provide feedback. The recorded presentation and associated survey were available online for 4 months after the conference. The ASPE Executive Committee approved the final manuscript.

## Results

All interested participants had experience as a PETA educator/trainer and/or PETA program administrator. Nineteen of the 32 interested individuals were selected as panelists. Thirteen individuals completed all three rounds of the survey (Tables 2 and 3).

**Table 2:** Survey completion

Interested participants	32
Qualified applicants	25
Completed round 1	19
Completed round 2	13
Completed round 3	13

**Table 3:** Panelists

Robert Bolyard (United States)
Sergio Bozzo (Chile)
Valerie Fulmer (United States)
Gail Furman (United States)
Karen Lewis (United States)
Kevin Kingston (United States)
Teresa Sapieha-Yanchak (United States)
Nicola Ngiam Siew Pei (Singapore)
Kris Slawinski (United States)
Lisa Steele (United States)
Francine Viret (Switzerland)
Marsha Yelen (United States)
Rose Zaeske (United States)

Statistics and summarized feedback are available in the Supplementary material. Minor revisions to language were made after the final survey, as noted below, so the Supplementary File does not reflect the final SOBP. No additional feedback was provided from the ASPE 2021 conference.

### Domains

The Practices included within this document met statistical consensus for applicability to PETA programs during the Delphi Process. Two items from the ASPE SOBP [5], both related to high-stakes assessment, did not reach consensus (Table 4). The lack of inclusion of a Practice or Principle does not diminish its importance within the ASPE SOBP [5] but does highlight the unique context of PETA programs and possibilities for future research. There were 12 additions beyond the original Practices within the ASPE SOBP. To support interoperability of the SOBP, the numbering of the Practices has been retained from the original ASPE SOBP [5], which is consistent with the ASPE GTA/MUTA SOBP [6]. Similar to the ASPE GTA/MUTA SOBP, common Practice modifications included revising “case” and “role portrayal” to “instruction” and “instructional session”, and “SP” to “PETA”. Additional revisions throughout the document reflect broader changes in the educational lexicon [32], such as revising terms (e.g., changing “stakeholder” to “relevant party” [33]).

**Table 4:** Practices from ASPE SOBP that did not meet consensus

3.4.7 In high stakes assessment, verify inter-rater reliability, in which a learner would achieve the same score when rated by different SPs
3.4.8 In high stakes assessment, verify intra-rater reliability, in which SPs would assign the same score to an identical performance at different points in time

### Domain 1: safe work environment

Physical and psychological safety are critical in all working and learning environments, but are especially crucial when instructing learners to conduct physical examinations with one’s body as PETAs do. PETAs provide feedback and instruction based on their experience receiving a physical examination from the learner, which is a unique teaching/learning interaction. SP Educators can work with PETAs to create a respectful atmosphere that emphasizes confidentiality and fosters bodily autonomy, meaning that the PETA is coached to have control of what they do with their body at all times [14, 32]. The SP Educator therefore should work with PETAs to determine reasonable practice limits on the number of examinations in a session [14, 34] and the time allotted for each physical exam. Including PETA input regarding their health and safety can strengthen program safety for all. Special care may be needed for PETAs with known pathology to reduce the risk of personal and physical challenges of participation [18]. There are three principles in Domain 1: safe work practices, confidentiality, and respect (Table 5).

**Table 5:** Domain 1

Principle	Practice
1.1 Safe Work Practices	1.1.1 Foster safe working conditions in the design of the activity (e.g., number of rotations, number of breaks, degree of physical contact and exposure, number of exam maneuvers performed daily, PETA feedback). 1.1.2 Anticipate and recognize potential occupational hazards, including threats to PETA safety in the environment (e.g., allergenic substances, exposure to sharps, air quality, live defibrillators.) 1.1.3 Screen PETAs to determine they are appropriate for the instructor role (e.g., no conflict of interest, no compromising of their psychological or physical safety). 1.1.4 Allow PETAs to opt out of any given activity if they feel it is not appropriate for them to participate. 1.1.5 Brief PETAs so they are clear about the guidelines and parameters of the session before booking or training. 1.1.6 Provide PETAs with strategies to mitigate potential adverse effects of the teaching activity and prevent physical injury or fatigue. 1.1.7 Inform relevant parties about the criteria and processes for terminating a session if they deem it harmful. 1.1.8 Structure time and create a process for debriefing. 1.1.9 Monitor for and respond to PETAs who have experienced adverse effects from participation in an activity. 1.1.10 Provide a process for relevant parties to report adverse effects from participation in a PETA activity (e.g., documentation and action steps to resolve the situation and provide closure). 1.1.11 Support PETAs who act in accordance with delineated program expectations if a complaint is made about them. 1.1.12 Manage client expectations of PETA possibilities and limitations. 1.1.13 Work with clients to clearly define the expected scope and limitation of PETA involvement in work assignments. 1.1.14 Define and provide clear limitations related to the specific skills that are permissible to instruct in a session (e.g., maximum number of exams, collection of samples, skills that are to be excluded from instruction). 1.1.15 Provide participants with a description of the nature of the instructional session before entering the room.
1.2 Confidentiality	1.2.1 Understand the specific principles of confidentiality that apply to all aspects of each session. 1.2.2 Ensure that PETAs understand and maintain the principles of confidentiality related to specific sessions. 1.2.3 Facilitate PETA’s understanding and maintenance of the principles of confidentiality related to specific sessions. 1.2.4 Protect the privacy of the personal and physical information of all relevant parties, including that which may be revealed within a session.
1.3 Respect	1.3.1 Respect PETAs’ self-identified boundaries (e.g., modesty, limits to physical touch, impact on person). 1.3.2 Provide PETAs with adequate information so that they can make informed decisions about participation in work assignments. 1.3.3 Provide PETAs with information about if and how they are being compensated before accepting work (e.g., may include payment for training and work time, travel expenses, food vouchers, gift cards).

### Domain 2: instructional session development

Curricular goals and objectives drive PETA instructional session development. Once the goals and objectives are developed, subject matter experts and SP Educators work together to design session content and activities. Many PETA programs exist within broader SP programs or simulation centers that have defined methods for learner interactions (e.g., underpinning learning theories, feedback, and debriefing style). To the extent possible, PETA instructional sessions should reflect these methods in order to provide a consistent learner experience and support psychological safety. The learner “should understand and expect when, where, and how” they will receive feedback. Delivering it "unexpectedly, especially if it is negative, almost always is met by an emotional reaction impeding the processing of the information” [35]. To support safety and mitigate the risk of injury, session length should reflect consideration of PETA and learner fatigue levels and the amount of material covered. Scheduling too many sessions in a row and/or covering too much material in one session could result in PETA injury.

PETA training materials are often derived from the learner’s physical examination instructional materials and are then supplemented with information on how PETAs can facilitate the sessions. For the sessions to be successful, materials should provide the PETAs with guidance for facilitating a supportive environment that incorporates feedback to help learners refine their physical examination skills and other associated clinical skills (e.g., draping, cultivating privacy, maintaining patient autonomy, providing anticipatory education). SP Educators need to schedule adequate time for the iterative process of developing and revising materials for PETAs. There are two Principles in Domain 2: preparation and instructional material components (Table 6).

**Table 6:** Domain 2

Principle	Practice
2.1 Preparation	2.1.1 Develop instructional materials that align with measurable learning objectives. 2.1.2 Identify and engage relevant PETA instructional material experts to assist in the creation of materials. Experts include members of the SP Program with expertise in PETA methodology. 2.1.3 Develop instructional materials that are based on up-to-date clinical practice guidelines and respect the individuals involved in the instructional session to avoid bias or stereotyping of marginalized populations. 2.1.4 Support an instructional material development process that allows sufficient time to draft, review, and edit instructional materials prior to implementation. 2.1.5 Address changes arising from dry-runs, or other piloting processes prior to implementation of the instructional materials.
2.2 Instructional material components	Develop instructional material components that include the following when appropriate: 2.2.1 Clear goals and objectives that can be addressed. 2.2.2 Goals and objectives that specify the intended level and type of learners. 2.2.3 Session design that meets the purpose. 2.2.4 Session design that is repeatable. 2.2.5 Information for PETAs includes description of technique/criteria for assessment that are written in clear and understandable terms. Information may include images and videos as indicated. 2.2.6 Training resources (e.g., props, videos, task trainers). 2.2.7 Physical exam specific feedback or debriefing guidelines. 2.2.8 Briefing instructions, time frames, instructions to learners. 2.2.9 Evaluation instruments and performance measures (e.g., checklists and rating scales, participant and facilitator evaluations). 2.2.10 Training protocols for raters (PETA or other). 2.2.11 Data for managing the documents and recruiting PETAs (e.g., author information, date of development, specific physical requirements).

### Domain 3: PETA training

PETA training addresses physical examination techniques and the effective provision of feedback. Educational goals and learning objectives will determine the degree of training necessary to learn these techniques and effectively provide feedback [36].

PETAs should receive training that allows them to identify the sensations of an appropriate/accurate physical examination to facilitate feedback. PETAs additionally benefit from the knowledge of their own anatomy and relevant examination findings, whether or not the PETA program selects PETAs with specific conditions [9, 12–14, 16, 18–20, 34, 37–44].

Effective and expected provision of formative feedback is also a component of PETA training. Formative feedback involves clarification of goals, engaging the learner in self-assessment and dialogue, providing instruction on how to modify the performance, and facilitating opportunities to close the gap between current and desired performance all while supporting the learner’s self-confidence [45]. There are five principles in Domain 3: preparation for training, training for teaching, training for feedback, training for completion of assessment instruments, and reflection on the training process (Table 7).

**Table 7:** Domain 3

Principle	Practice
3.1 Preparation for Training	3.1.1 Review the purpose, objectives and outcomes, logistics, and instructional materials of the activity. 3.1.2 Address one’s own knowledge gaps, if any. 3.1.3 Create a training plan that is responsive to the context and format of each activity (e.g., group training for standardization, video review, practice with physical examination equipment). 3.1.4 Gather training resources to supplement training. 3.1.5 Create clear written instructions informed by PETA feedback. 3.1.6 Consider that training could be modular, varied by content and/or training and/or learner preferences.
3.2 Training for teaching	3.2.1 Review with PETAs the key objectives, responsibilities, context (e.g., formative, level of learner, placement in curriculum), and format (e.g., length of session, number of exams) of each activity. 3.2.2 Engage PETAs in discussion and practice of examination techniques and of coaching/facilitation skills. 3.2.3 Provide PETAs with strategies to deal with unanticipated learner questions and behaviors. 3.2.4 Facilitate consistency and accuracy of examination techniques of individual PETAs, and among groups of PETAs teaching the same material. 3.2.5 Facilitate PETA readiness for teaching activity through repeated practice and targeted feedback. 3.2.6 Provide periodic refresher or re-calibration training, even if the instructional session does not change. 3.2.7 Include a screening examination provided by a healthcare provider or qualified trainer to demonstrate the sensations the PETA may experience during an instructional session as well as to identify variations from normal anatomy or physiology. 3.2.8 Provide pre-session learner resources to PETAs to prepare them for the instructional sessions (e.g., institution-prepared materials, textbook reading assignments). 3.2.9 Provide PETAs with the ability to demonstrate proficiency in examination maneuvers that they will be instructing or assessing.
3.3 Training for feedback	3.3.1 Review with PETAs the fundamental principles of feedback as they relate to the planned activity. 3.3.2 Inform PETAs of the feedback objectives and level of the learners with whom they will be working. 3.3.3 Inform PETAs of the feedback logistics and setting (e.g., one-on-one feedback with learner, small group feedback). 3.3.4 Train PETAs to use their observations, responses, and knowledge to provide feedback on observable, modifiable behaviors in learners. 3.3.5 Facilitate PETA readiness through repeated practice and targeted feedback. 3.3.6 Train PETAs to utilize communication techniques that optimize learning outcomes during instructional sessions (e.g., correction of technique, use of inquiry, avoidance of leading questions). 3.3.7 Review methods of promoting instructional effectiveness through immediate feedback.
3.4 Training for completion of assessment instruments	3.4.1 Develop PETA’s understanding of the nature, context, and objectives of the assessment. 3.4.2 Develop PETA’s understanding of the format of the assessment instrument. 3.4.3 Determine that PETAs are able to complete assessment instruments in the time allotted. 3.4.4 Provide PETAs with practice completing assessment instruments with a variety of learner behaviors. 3.4.5 Develop PETA’s understanding of both the principles and receptive experiences of any physical exam maneuvers they will be assessing. 3.4.6 Facilitate consistent and accurate completion of an assessment instrument by individual PETAs, and among groups of PETAs performing the same task.
3.5 Reflection on the training process	3.5.1 Reflect on one’s own training practices for future improvement (e.g., evaluation forms, debriefing, video review).

### Domain 4: program management

Program management is complex with many intersecting considerations. PETA programs provide an expert/educator in PETA methodology, often the SP Educator, who trains individuals to teach physical examinations to designated learners. Programs work to develop collaborative expertise in using PETA methodology with other relevant parties (e.g., clinicians, PETAs). PETA programs are often part of an SP program but may be part of a unique organization or system. Regardless of location, PETA programs administer PETA services and create and execute policies and procedures that contribute to and guide this specific educational environment and experience for learners. Program responsibilities include planning logistics for instructional sessions; recruiting and training PETAs; conducting quality review and improvement; and creating financial management, business, and strategic plans that are efficient and cost-effective. PETA programs uphold institutional and professional standards of practice. Clearly stated policies and procedures demonstrate that a PETA program meets ethical, humanistic, legislated, institutional, and professional practice standards for screening, recruiting, managing, and monitoring PETAs and other staff working with them. There are six principles in Domain 4: purpose, expertise, policies and procedures, records management, team management, quality management (Table 8).

**Table 8:** Domain 4

Principle	Practice
4.1 Purpose	4.1.1 Articulate a mission statement for the program. 4.1.2 Develop program goals. 4.1.3 Identify measurable objectives for each goal (where applicable).
4.2 Expertise	4.2.1 Possess a depth of knowledge in PETA methodology. 4.2.2 Advocate for the integration of PETA methodology into the curriculum where appropriate. 4.2.3 Identify when PETA methodology should be incorporated into an instructional session. 4.2.4 Collaborate with subject matter experts to design instructional sessions, training, and assessment materials. 4.2.5 Train PETAs according to instructional session or project parameters.
4.3 Policies and procedures	4.3.1 Develop and document policies to guide program activities. 4.3.2 Develop and document policies that take into consideration disability access and inclusion. 4.3.3 Develop and document business processes and procedures, including but not limited to creating financial management, business, and strategic plans. 4.3.4 Keep policies and procedures current and accessible. 4.3.5 Distribute policies and procedures to relevant parties. 4.3.6 Develop and document policies and procedures to guide responses to a learner-identified unexpected finding. 4.3.7 Engage PETAs in developing and reviewing PETA program policies and procedures.
4.4 Records management	4.4.1 Collaborate with subject matter experts to develop a system for reporting learner performance to relevant parties (e.g., learners, curriculum developers, faculty, administration). 4.4.2 Develop policies for instructional material sharing and archiving. 4.4.3 Develop and document methods for securely storing, archiving, and destroying confidential data (e.g., PETA records, learner data, video data, consent forms, release forms).
4.5 Team management	4.5.1 Consult with legal, financial, and human resources experts to determine that the status of PETAs (e.g., employee, independent contractor, volunteer) and compensation structure (if applicable) comply with institutional requirements. 4.5.2 Develop processes to identify, screen, interview, select, debrief, and maintain PETAs and staff. 4.5.3 Recruit and maintain a cohort of PETAs that reflects the diversity of the people in the community. 4.5.4 Establish policies and procedures for the psychological, physical, and environmental safety of PETAs, learners, staff, and faculty. 4.5.5 Advocate for ongoing professional development opportunities for all staff, including PETAs.
4.6 Quality management	4.6.1 Gather data regularly to assess the alignment of program activities with legislated, institutional, and program policies and procedures. 4.6.2 Gather feedback regularly from PETAs, learners, faculty, and other relevant parties regarding the quality of services provided by the program. 4.6.3 Analyze data and other feedback in a timely manner. 4.6.4 Implement changes for continuous improvement. 4.6.5 Inform relevant parties of changes made based on their feedback. 4.6.6 Provide the opportunity for PETAs to engage in self-evaluation.

### Domain 5: professional development

PETAs partner with healthcare educators to facilitate the learning of current and future healthcare professionals. PETAs in turn experience learning and development with existing healthcare professionals as they prepare for their instructional sessions. So, too, must SP Educators learn and develop as they instruct, train, guide, and administrate PETAs for events and/or instructional sessions within existing curricula. As with the original ASPE SOBP [5], Steinert’s [46] model of faculty development informs our definitions of professionalism and professional development as it relates to our context. There are three Principles in Domain 5: career development, scholarship, and leadership (Table 9).

**Table 9:** Domain 5

Principle	Practice
5.1 Career development	5.1.1 Develop and promote expertise in knowledge, skills, and attitudes related to PETA instructional sessions. 5.1.2 Develop and promote expertise in theories, principles, and processes of education and assessment relevant to the context of one’s practice (e.g., medical education, nursing education). 5.1.3 Maintain membership in professional simulation societies (e.g., ASPE, ASPiH, INACSL, SESAM, SSH) or medical education societies. 5.1.4 Engage in educational opportunities (e.g., professional conferences, courses, degree programs, certifications). 5.1.5 Develop personal management skills (e.g., time management, wellness strategies, career planning). 5.1.6 Seek out opportunities for career mentoring.
5.2 Scholarship	5.2.1 Develop an understanding of the range of opportunities for scholarship in PETA methodology. 5.2.2 Identify and/or develop new contexts for PETA methodology. 5.2.3 Contribute to the evolution of best practices through innovation, research, and dissemination of emerging methods in various venues (e.g., publications, presentations).
5.3 Leadership	5.3.1 Promote understanding and development of PETA methodology locally, nationally, and internationally. 5.3.2 Mentor and support PETAs and other SP Educators within one’s institution and within the community of practice. 5.3.3 Seek out and advocate for the growth of leadership skills (e.g., collaboration, team building, change management, interpersonal effectiveness, conflict resolution).

## Discussion

Using the Delphi process, ASPE engaged 13 PETA methodology experts from four countries to develop the ASPE PETA SOBP. Only two items from the ASPE SOBP [5], both related to high-stakes assessment, did not reach consensus (3.4.7 and 3.4.8). There were 12 additions beyond the original ASPE SOBP Practices, reflecting the uniqueness of PETA methodology compared to the broader SP methodology. Some of the additional Practices were similar to those added to the GTA/MUTA SOBP [6] (1.1.14, 1.1.16, 3.2.6, 3.2.8, 3.2.9), highlighting similarities between the PETA and GTA/MUTA methodology. Other Practices in the PETA SOBP but not the GTA/MUTA SOBP (2.2.9, 2.2.10, 2.2.11, 3.2.7, 4.2.4, 4.2.5, 4.3.3, 4.3.6, Domain 5) underline the potential differences in the work of PETAs and GTAs/MUTAs. Comparing the PETA SOBP with the GTA/MUTA SOBP highlights potential differences in the role of the SP Educator engaging in the different programs. Further research should explore the similarities and differences between PETA and GTA/MUTA methodologies, as well as how they are positioned within broader SP methodology. Research questions may include:

- How does the SP Educator role vary when working with PETAs, GTA/MUTAs, and SPs?
- What are the similarities and differences in PETA, GTA, and MUTA instructional sessions?
- Is there a difference in how PETA programs and GTA/MUTA programs are integrated into SP programs?
- How does the type of physical examination (e.g., musculoskeletal exam vs pelvic exam) being instructed impact perception of risk to physical and psychological safety for relevant parties?

Currently, having a distinct PETA SOBP is important to promote a comprehensive and holistic approach to PETA methodology and to enable SP Educators to support PETAs in their work.

We propose that future research on the SP Educator’s experience in PETA and GTA/MUTA programs take place before future revisions of the ASPE PETA SOBP and the ASPE GTA/MUTA SOBP [6]. This work will help determine whether the SP Educator role is sufficiently aligned in PETA and GTA/MUTA programs such that a single set of standards could apply to all programs engaging in physical examination instruction. ASPE will continue to maintain SOBP for PETA, SP, and GTA/MUTA programs.

The limited publications on PETA-specific methodology [7] may indicate that PETA programs are either uncommon or not recognized as PETA programs (perhaps through their placement within broader SP programs). The terminology used to describe the PETA role is variable [7], but whatever terms are used, programs engaging with this unique application of SP methodology should be administered by an SP Educator who is guided by the PETA SOBP to ensure appropriate engagement with PETAs. For example, although many SPs engage in feedback related to simulation [47, 48], the feedback that PETAs provide in an instructional session is iterative and formative [45]. This kind of feedback may differ from what SPs are used to providing, and PETAs should be carefully supported to undertake this task. In all contexts, the SP Educator is responsible for identifying and reasonably applying the appropriate standards for their program type (PETA, SP, GTA/MUTA).

## Limitations

Although we intended to include diverse international perspectives, panelists from only four countries completed all three rounds of surveys (Chile, Singapore, Switzerland, USA). The literature search demonstrates additional publications from Australia, Canada, England, and Germany, although some of these publications are 20 or more years old. Future iterations of the ASPE PETA SOBP should identify specific PETA methodology leaders within these and other countries to facilitate a more diverse perspective.

Survey timing during the COVID-19 pandemic may have impacted participation. Sixty-eight percent of participants who completed the Round 1 survey completed all rounds of the Delphi Process. In comparison, 79% of individuals who started the GTA/MUTA SOBP completed the Delphi Process [6]. We suspect that the higher rate of attrition and the lack of feedback from the ASPE conference presentation were related to the COVID-19 pandemic and the virtual nature of the conference.

Two members of the research team (KL and MY) completed the surveys for the Delphi process, so there could have been bias introduced through their involvement. However, they did not have access to participant responses before they completed the surveys. All survey results were anonymous, and feedback to participants was generalized to mitigate bias.

Some textbooks [49] detail implementation strategies for PETA programs, but their inclusion was beyond the scope of our literature search.

## Conclusion

The ASPE PETA SOBP identifies expert consensus for aspirational standards for all PETA programs; individual application will vary based on local context. The PETA SOBP is critical to ensuring high-quality learning opportunities that are physically and psychologically safe for PETAs, learners, and their future patients while decreasing an institution’s legal risk. ASPE will continue to develop and promote a rigorous methodology to reflect the growth of the role of SP Educators and PETAs as well as expert consensus in the field in the ASPE PETA SOBP.

## Supplementary Information

Supplementary Material 1.

## Data Availability

A summary of data generated or analyzed during this study is included in a supplementary information file. The datasets analyzed during the current study are available from the corresponding author upon reasonable request.

## Abbreviations

ASPE: Association of SP Educators
GTA: Gynecological Teaching Associate
MUTA: Male Urogenital Teaching Associate
PETA: Physical Examination Teaching Associate
SOBP: Standards of Best Practice
SP: Standardized/simulated patient/participant
