# Supplementary material for: Association of SP Educators (ASPE) Physical Examination Teaching Associate (PETA) Standards of Best Practice (SOBP)
Source: Adv Simul (Lond). 2026 Feb 19;11:12. doi: 10.1186/s41077-025-00373-z (PMC12922199; doi:10.1186/s41077-025-00373-z)
Supplement: Supplementary file 1 — Supplementary Material 1. [file 41077_2025_373_MOESM1_ESM.pdf]

Online Supplemental File
ASPE PETA SOBP Final Survey Results

	Not Important/ Applicable (1)	Minimally Important (2)	Somewhat Important (3)	Very Important (4)	Critically Important (5)
	**Domain 1: Safe Work Environment**				

	ROUND 1 RESULTS	ROUND 2 RESULTS	ROUND 3 RESULTS
**1.1 Safe Work Practices**			
1.1.1. Ensure safe working conditions in the design of the activity (e.g., number of rotations, number of breaks, physical, cognitive, and psychological challenges in the role portrayal).	Mean (SD): 4.95 (.22)<br>Results: Consensus achieved.<br>"Ensure safe working conditions in the design of the activity (e.g., number of rotations, number of breaks, degree of physical contact and exposure, physical, cognitive, and psychological challenges in the role portrayal, PETA feedback)."<br>Comments: Consider including PETA feedback on the design of the activity, and degree of physical contact and exposure.	Reached consensus in Round 1.<br>"Ensure safe working conditions in the design of the activity (e.g., number of rotations, number of breaks, degree of physical contact and exposure, number of exam maneuvers performed daily, physical, cognitive, and psychological challenges in the role portrayal, PETA feedback)."<br>Comments: Additional example provided based on feedback regarding other Principles.	
1.1.2 Anticipate and recognize	Mean (SD): 4.75 (.44)	Reached consensus in	

potential occupational hazards, including threats to SP safety in the environment (e.g., allergenic substances, exposure to sharps, air quality, live defibrillators).	Results: Consensus achieved. “Anticipate and recognize potential occupational hazards, including threats to PETA safety in the environment (e.g., allergenic substances, exposure to sharps, air quality, live defibrillators).”	Round 1.	
1.1.3 Screen SP to ensure that they are appropriate for the role (e. g., no conflict of interest, no compromising of their psychological or physical safety).	Mean (SD): 4.55 (.51) Results: Consensus achieved. “Screen PETAs to ensure that they are appropriate for the instructor role (e. g., no conflict of interest, no compromising of their psychological or physical safety).”	Reached consensus in Round 1.	
1.1.4. Allow SPs to opt out of any given activity if they feel it is not appropriate for them to participate.	Mean (SD): 4.80 (.41) Results: Consensus achieved. “Allow PETAs to opt out of any given activity if they feel it is not appropriate for them to participate.”	Reached consensus in Round 1.	
1.1.5 Brief SPs so they are clear about the guidelines and parameters of a simulation activity.	Mean (SD): 4.75 (.44) Results: Consensus achieved. “Brief PETAs so they are clear about the guidelines and parameters of the session before booking or training.”	Reached consensus in Round 1.	

	Comments: Consider including briefing before booking or training.		
1.1.6 Provide SPs with strategies to mitigate potential adverse effects of role portrayal and prevent physical injury or fatigue	Mean (SD): 4.80 (.41)<br>Results: Consensus achieved.<br>“Provide PETAs with strategies to mitigate potential adverse effects of the teaching activity and prevent physical injury or fatigue.”	Reached consensus in Round 1.	
1.1.7 Inform SPs and clients about the criteria and processes for terminating a simulation if they deem it harmful.	Mean (SD): 4.60 (.75)<br>Results: Consensus achieved.<br>“Inform PETAs and clients about the criteria and processes for terminating a session if they deem it harmful.”	Reached consensus in Round 1.	
1.1.8 Structure time and create a process for de-roling and/or debriefing.	Mean (SD): 4.32 (.82)<br>Results: Consensus achieved.<br>“Structure time and create a process for debriefing.”	Reached consensus in Round 1.	
1.1.9 Monitor for and respond to SPs who have experienced adverse effects from participation in an activity.	Mean (SD): 4.80 (.52)<br>Results: Consensus achieved.<br>“Monitor for and respond to PETAs who have experienced adverse effects from participation in an activity.”	Reached consensus in Round 1.	
1.1.10 Provide a process for SPs and clients to report adverse effects from	Mean (SD): 4.75 (.44)<br>Results: Consensus achieved.	Reached consensus in Round 1.	

participation in a SP activity (e.g., documentation and action steps to resolve the situation).	"Provide a process for PETAs and clients to report adverse effects from participation in a PETA activity (e.g., documentation and action steps to resolve the situation and provide closure)." Comments: Consider adding "and closing the loop" to the e.g. list.		
1.1.11 Support SPs who act in accordance with delineated program expectations if a complaint is made about them.	Mean (SD): 4.75 (.44) Results: Consensus achieved. "Support PETAs who act in accordance with delineated program expectations if a complaint is made about them."	Reached consensus in Round 1.	
1.1.12 Manage client expectations of a SPs possibilities and limitations.	Mean (SD): 4.70 (.57) Results: Consensus achieved. "Manage client expectations of a PETAs possibilities and limitations."	Reached consensus in Round 1.	
1.1.13 Work with clients to clearly define the expected scope of SP involvement in work assignments.	Mean (SD): 4.70 (.57) Results: Consensus achieved. "Work with clients to clearly define the expected scope and limitation of PETA involvement in work assignments."	Reached consensus in Round 1.	
**New Additions**			

	1.1.14 "Define and provide clear limitations to the specific skills that are permissible to instruct in a session (e.g. maximum number of exams, collection of samples, skills that are to be excluded from instruction)."	Mean (SD): 4.75 (.45)<br>Results: Consensus achieved.<br>Comments: PETAs should be consulted regarding limitations. PETAs should be empowered to end a session as necessary (addressed in 1.1.7).	Reached consensus in Round 2.
	1.1.15 "Ensure acknowledgement of participants that they are aware of the nature of the instructional session prior to entering the room."	Mean (SD): 4.67 (.65)<br>Results: Consensus achieved.<br>"Ensure participants are aware of the nature of the instructional session prior to entering the room."<br>Comments: Revised for clarity that written acknowledgement is not required.	Reached consensus in Round 2.
**1.2 Confidentiality**			
1.2.1 Understand the specific principles of confidentiality that apply to all aspects of each simulation event.	Mean (SD): 4.70 (.47)<br>Results: Consensus achieved.<br>"Understand the specific principles of confidentiality that apply to all aspects of each session."	Reached consensus in Round 1.	
1.2.2 Ensure that SPs understand and maintain the principles of confidentiality related to specific	Mean (SD): 4.80 (.41)<br>Results: Consensus achieved.<br>"Ensure that PETAs	Reached consensus in Round 1.	

simulation events.	understand and maintain the principles of confidentiality related to specific sessions.”		
1.2.3 Protect the privacy of the personal information of all stakeholders, including that which may be revealed within a simulation activity.	Mean (SD): 5.00 (.00)<br>Results: Consensus achieved.<br>“Protect the privacy of the personal and physical information of all stakeholders, including that which may be revealed within a session.”<br>Comments: Consider adding privacy of personal and physica*l* information.	Reached consensus in Round 1.	
**1.3 Respect**			
1.3.1 Respect SPs’ self-identified boundaries (e.g., modesty, limits to physical touch, impact on person).	Mean (SD): 4.79 (.42)<br>Results: Consensus achieved.<br>“Respect PETAs’ self-identified boundaries (e.g., modesty, limits to physical touch, impact on person).”	Reached consensus in Round 1.	
1.3.2 Provide SPs with adequate information so that they can make informed decisions about participation in work assignments.	Mean (SD): 4.90 (.31)<br>Results: Consensus achieved.<br>“Provide PETAs with adequate information so that they can make informed decisions about participation in work assignments.”	Reached consensus in Round 1.	
1.3.3 Ensure that SPs understand if and how they are being compensated	Mean (SD): 4.65 (.49)<br>Results: Consensus achieved.	Reached consensus in Round 1.	

before accepting work (e.g., may include payment for training and work time, travel expenses, food vouchers, gift cards).	"Ensure that PETAs understand if and how they are being compensated before accepting work (e.g., may include payment for training and work time, travel expenses, food vouchers, gift cards)."		

	Not Important/ Applicable (1)	Minimally Important (2)	Somewhat Important (3)	Very Important (4)	Critically Important (5)
**Domain 2: Instructional Material Development**					

	ROUND 1 RESULTS	ROUND 2 RESULTS	ROUND 3 RESULTS
**2.1 Preparation**			
2.1.1 Ensure that cases align with measurable learning objectives.	Mean (SD): 4.52 (.96)<br>"Ensure that instructional materials align with the measurable learning objectives."<br>Comments: Consider changing name of standard to reflect intention of PETA session.	Mean (SD): 4.75 (.45)<br>Results: Consensus achieved.<br>Comments: Change name of Domain to Instructional Material Development. Faculty and instructors should be familiar with learning objectives.	Reached consensus in Round 2.
2.1.2 Identify and engage relevant subject matter experts to assist in the creation of materials.	Mean (SD): 4.63 (.50)<br>Results: Consensus achieved.<br>"Identify and engage relevant PETA instructional material experts to assist in the creation of materials. Experts should include members of the SP Program."<br>Comments: Consider that experts in areas of curriculum and SP simulation programs may contribute to PETA material development	Reached consensus in Round 1.	

2.1.3 Ensure that cases are based on authentic problems and respect the individuals represented in a case to avoid bias or stereotyping marginalized populations.	Mean (SD): 4.26 (1.04)<br>Results: "Ensure that instructional materials are based on authentic problems and respect the individuals represented in a case to avoid bias or stereotyping marginalized populations."<br>Comments: PETA activities are focused on teaching and assessment, not case-based histories.	Mean (SD): 4.77 (0.44)<br>Results: Consensus achieved. "Ensure that instructional materials are based on up-to-date clinical practice guidelines and respect the individuals involved in the instructional session to avoid bias or stereotyping marginalized populations."<br>Comments: PETAs do not typically portray cases, they instruct learners.	Reached consensus in Round 2.
2.1.4 Ensure that case development process allows sufficient time to draft, review, and edit case materials prior to implementation.	Mean (SD): 4.31 (.67)<br>Results: Consensus achieved. "Ensure that instructional material development process allows sufficient time to draft, review, and edit case materials prior to implementation."<br>Consider replacing "case" with training/teaching material.	Reached consensus in Round 1.	
2.1.5 Ensures that changes arising from dry-runs, or other piloting processes are addressed prior to implementation of the case.	Mean (SD): 4.37 (.50)<br>Results: Consensus achieved. "Ensures that changes arising from dry-runs, or other piloting processes are addressed prior to implementation of the instructional materials."<br>Comments: Consider replacing	Reached consensus in Round 1.	

	"case" with training/teaching material.		
**2.2 Case Components. Ensure case components include the following when appropriate:** "Instructional Material Components. Ensure instructional material components include the following when appropriate:" Consider replacing "case" with training/teaching material.			
2.2.1 Clear goals and objectives that can be addressed.	Mean (SD): 4.63 (.50) Results: Consensus achieved. "Clear goals and objectives that can be addressed."	Reached consensus in Round 1.	
2.2.2 Goals and objectives that specify the intended level of learners.	Mean (SD): 4.42 (.77) Results: Consensus achieved. "Goals and objectives that specify the intended level and type of learners." Comments: Consider adding type of learner.	Reached consensus in Round 1.	
2.2.3 Simulation design that meets the purpose.	Mean (SD): 4.74 (.45) Results: Consensus achieved. "Session design that meets the purpose." Comments: Consider replacing simulation term with teaching activity design.	Reached consensus in Round 1.	
2.2.3 Simulation design that is repeatable.	Mean (SD): 4.73 (.45) Results: Consensus achieved. "Session design that is repeatable." Comments: Consider replacing simulation term with teaching	Reached consensus in Round 1.	

	activity design.		
2.2.5 Information for SPs (e.g., situation and backstory, history, affect and demeanor, signs and symptoms to simulate, cues).	Mean (SD): 4.12 (1.29)<br>"Information for PETAs includes description of technique, written in clear and understandable terms."<br>Comments: Consider change to information includes description/criteria for assessment that are written in clear and understandable terms and change SP/GTA/MUTA to PETA.	Mean (SD): 4.75 (.45)<br>Results: Consensus achieved.<br>"Information for PETAs includes description of technique/criteria for assessment that are written in clear and understandable terms Information may include images and videos as indicated."<br>Comments: Images and videos may provide additional clarity.	Reached consensus in Round 2.
2.2.6 Training resources (e.g., props, moulage, videos, task trainers).	Mean (SD): 4.47 (.70)<br>Results: Consensus achieved. No additional comments.<br>"Training resources (e.g., props, moulage, videos, task trainers)."	Reached consensus in Round 1.	
2.2.7 Case-specific feedback or debriefing guidelines.	Mean (SD): 4.58 (.61)<br>Results: Consensus achieved.<br>"Physical exam specific feedback or debriefing guidelines."<br>Consider replacing case-specific to more relevant terms.	Reached consensus in Round 1.	
2.2.8 Briefing instructions, time frames, instructions to learners.	Mean (SD): 4.58 (.51)<br>Results: Consensus achieved. No additional comments.<br>"Briefing instructions, time frames, instructions to learners."	Reached consensus in Round 1.	
2.2.9 Evaluation instruments	Mean (SD): 4.58 (.61)	Reached consensus in Round 1.	

and performance measures (e.g., checklists and rating scales, participant and facilitator evaluations).	Results: Consensus achieved. No additional comments.<br>"Evaluation instruments and performance measures (e.g., checklists and rating scales, participant and facilitator evaluations)."		
2.2.10 Training protocols for raters (SP or other).	Mean (SD): 4.63 (.50)<br>Results: Consensus achieved.<br>"Training protocols for raters (PETA or other)."<br>Comments: Change SP/GTA/MUTA to PETA	Reached consensus in Round 1.	
2.2.11 Data for managing the documents and recruiting SPs (e.g., author information, date of development, patient demographics, body type criteria).	Mean (SD): 4.21 (.54)<br>Results: Consensus achieved.<br>"Data for managing the documents and recruiting PETAs (e.g., author information, date of development, patient demographics, body type criteria)."<br>Comments: Change SP/GTA/MUTA to PETA	Reached consensus in Round 1.	

	Not Important/ Applicable (1)	Minimally Important (2)	Somewhat Important (3)	Very Important (4)	Critically Important (5)
**Domain 3: PETA Training**					

	ROUND 1 RESULTS	ROUND 2 RESULTS	ROUND 3 RESULTS
**3.1 Preparation for Training**			
3.1.1 Review the purpose, objectives and outcomes, logistics, and case materials of the activity.	Mean (SD): 4.78 (.54)<br>Results: Consensus achieved. “Review the purpose, objectives and outcomes, logistics, and instructional materials of the activity.”	Reached consensus in Round 1.	
3.1.2 Address one’s own knowledge gaps, if any.	Mean (SD) 4.67 (.59)<br>Results: Consensus achieved. Knowledge gaps are unavoidable, consult experts.	Reached consensus in Round 1.	
3.1.3 Create a training plan that is responsive to the context and format of each activity (e.g., group training for standardization, video review, practice with simulation equipment).	Mean (SD): 4.67 (.49)<br>Results: Consensus achieved. Accommodating learning preferences & groups sizes	Reached consensus in Round 1.	
3.1.4 Gather training resources to supplement training.	Mean (SD): 4.39 (.61)<br>Results: Consensus achieved. May include videos, photos, and/or experts.	Reached consensus in Round 1.	

3.1.5 Gather administration documents and special instructions.	Mean (SD): 4.22 (.94)<br>Comments: Special instructions should be verbal and written	Mean (SD): 4.42 (.67)<br>Results: Consensus achieved. "Create clear written instructions informed by PETA feedback." Revised for consistent grammar with other practices.	Reached consensus in Round 2.
**New Additions**			
	3.1.6 "Structure training to avoid cognitive overload."<br>Comments: New PETA training should allow for absorption of the material.	Mean (SD):4.25 (.75)<br>Results: Consensus achieved. "Consider that training could be modular, varied by content and/ or training and / or learner preferences." Revised for consistent grammar with other practices.	Reached consensus in Round 2.
**3.2 Training for Teaching**			
3.2.1 Review with SPs the key objectives, responsibilities, context (e.g., formative, summative, level of learner, placement in curriculum) and format (e.g., length of encounter, type of encounter) of each activity.	Mean (SD): 4.72 (.57)<br>Results: Consensus achieved. Call for more specific administrative content. Suggested revision: "Review with PETAs the key objectives, responsibilities, context (e.g., formative, summative, level of learner, placement in curriculum) and format (e.g., length of encounter, type of encounter) of each activity."	Reached consensus in Round 1.	

3.2.2 Engage SPs in discussion and practice of role portrayal features (e.g., affect, signs and symptoms, behaviors).	Mean (SD): 4.11 (1.08)<br>Comments: Symptom portrayal not a key feature of PETA work. Consider: “Engage PETAs in discussion and practice of examination techniques and of coaching/facilitation skills.”	Mean (SD): 4.58 (.67)<br>Results: Consensus achieved. Coaching / facilitation skills should include discussion of appropriate and inappropriate behavior.	Reached consensus in Round 2.
3.2.3 Provide SPs with strategies to deal with unanticipated learner questions and behaviors.	Mean (SD): 4.61 (.61)<br>Results: Consensus achieved. No additional comments.<br>Suggested Revision: “Provide PETAs with strategies to deal with unanticipated learner questions and behaviors.”	Reached consensus in Round 1.	
3.2.4 Ensure consistency and accuracy of role portrayal of individual SPs, and among groups of SPs portraying the same role.	Mean (SD): 4.44 (.62)<br>Results: Consensus achieved.<br>Comments: Consistency is key / not “portrayal”. Consider “Ensure consistency and accuracy of examination techniques of individual PETAs, and among groups of PETAs teaching the same material.”	Reached consensus in Round 1.	
3.2.5 Ensure SP readiness for the simulation activity through repeated practice and targeted feedback.	Mean (SD): 4.56 (.62)<br>Results: Consensus achieved.<br>Comments: Emphasis on readiness for teaching / mention of higher stakes regarding institutional credibility.<br>Consider: “Ensure PETA	Reached consensus in Round 1.	

	readiness for teaching activity through repeated practice and targeted feedback."		
**New Additions**			
	3.2.6 "Provide periodic refresher or re-calibration training, even if the instructional session does not change."<br>Comments: PETAs should have scheduled reviews to ensure maintenance of teaching competencies.	Mean (SD): 4.25 (.87)<br>Results: Consensus achieved.	Reached consensus in Round 2.
	3.2.7 "A screening examination provided by a healthcare provider may be used to demonstrate the sensations the PETA may experience during an instructional session as well as to identify variations from normal anatomy or physiology."<br>Comments: PETAs should be educated on their own body including expected and unexpected findings both to enhance their ability to instruct and to provide clarification for learners.	Mean (SD): 4.25 (.97)<br>Results: Consensus achieved.<br>"Include a screening examination provided by a healthcare provider or qualified trainer to demonstrate the sensations the PETA may experience during an instructional session as well as to identify variations from normal anatomy or physiology."	Reached consensus in Round 2.
	3.2.8 "Ensure pre-session resources are provided to learners to prepare them for the	Mean (SD): 5.00 (0)<br>Results: Consensus achieved.<br>Comments: Necessity of	Reached consensus in Round 2.

	teaching sessions (ex. institution-prepared materials, textbook reading assignments, etc.).”<br>Comments: Consistent expectations for exam performance are important for learners.	resources sometimes determined by complexity of the event.	
	3.2.9 “Provide PETAs with the ability to demonstrate proficiency in examination maneuvers that they will be instructing or assessing.”<br>Comments: Demonstration of training efficacy, including variations on approach to techniques, should be considered prior to working with learners.	Mean (SD): 4.42 (.90)<br>Results: Consensus achieved.<br>Comments: Demonstrating a technique as a learner is different from demonstrating a technique as a PETA.	Reached consensus in Round 2.
**3.3 Training for Feedback**			
3.3.1 Review with SPs the fundamental principles of feedback as they relate to the planned activity.	Mean (SD): 4.67 (.59)<br>Results: Consensus achieved.<br>“Review with PETAs the fundamental principles of feedback as they relate to the planned activity.”<br>Comments: Feedback might be more important than content.	Reached consensus in Round 1.	
3.3.2 Inform SPs of the feedback objectives and level of the learners with whom	Mean (SD): 4.7222 (.46)<br>Results: Consensus achieved.<br>“Inform PETAs of the feedback	Reached consensus in Round 1.	

they will be working.	objectives and level of the learners with whom they will be working.”		
3.3.3 Inform SPs of the feedback logistics and setting (e.g., one-on-one feedback with learner, small group feedback, simulation debrief).	Mean (SD): 4.6667 (.77)<br>Results: Consensus achieved.<br>“Inform PETAs of the feedback logistics and setting (e.g., one-on-one feedback with learner, small group feedback, simulation debrief).”<br>Comments: Feedback might be more important than content.	Reached consensus in Round 1.	
3.3.4 Train SPs to use their observations, responses, and knowledge to provide feedback on observable, modifiable behaviors in learners.	Mean (SD): 4.8333 (.38)<br>Results: Consensus achieved.<br>“Train PETAs to use their observations, responses, and knowledge to provide feedback on observable, modifiable behaviors in learners.”	Reached consensus in Round 1.	
3.3.5 Ensure SP readiness through repeated practice and targeted feedback.	Mean (SD): 4.67 (.59)<br>Results: Consensus achieved.<br>“Ensure PETA readiness through repeated practice and targeted feedback.”	Reached consensus in Round 1.	
**New Additions**			
	3.3.6 “Train PETAs to utilize communication techniques that optimize learning outcomes during instructional sessions (e.g., correction of technique, use	Mean (SD): 4.67 (.49)<br>Results: Consensus achieved.<br>Comments: As instructors, PETAs must know how to provide appropriate feedback.	Reached consensus in Round 2.

	of inquiry, avoidance of leading questions, etc).”<br>Comments: As instructors, PETAs should be taught to teach.		
	3.3.7 "Review methods of promoting instructional effectiveness through real-time feedback."<br>Comments: As instructors, PETAs should be taught to teach.	Mean (SD): 4.25 (.754)<br>Results: Consensus achieved.<br>Comments: Consider who is giving and / or receiving the feedback.	Reached consensus in Round 2.
**3.4 Training for Completion of Assessment Instruments**			
3.4.1 Ensure that SPs understand the nature, context, and objectives of the assessment.	Mean (SD): 4.56 (.51)<br>Results: Consensus achieved.<br>“Ensure that PETAs understand the nature, context, and objectives of the assessment.”	Reached consensus in Round 1.	
3.4.2 Ensure that SPs understand the format of the assessment instrument.	Mean (SD): 4.56 (.62)<br>Results: Consensus achieved.<br>“Ensure that PETAs understand the format of the assessment instrument.”	Reached consensus in Round 1.	
3.4.3 Ensure that SPs are able to complete assessment instruments in the time allotted.	Mean (SD): 4.72 (.46)<br>Results: Consensus achieved.<br>“Ensure that PETAs are able to complete assessment instruments in the time allotted.”<br>Comment: Suggestion for administrative support.	Reached consensus in Round 1.	
3.4.4 Provide SPs with practice completing assessment	Mean (SD): 4.2778 (.89)<br>Results: Consensus achieved.	Reached consensus in Round 1.	

instruments with a variety of learner behaviors.	"Provide PETAs with practice completing assessment instruments with a variety of learner behaviors.<br>Comments: Clear expectations, practice, and experience foster a conducive learning environment/not applicable to all cases.		
3.4.5 Ensure that SPs understand both the principle and receptive experiences of any physical exam maneuvers they will be assessing.	Mean (SD): 4.44 (1.10)<br>Results: "Ensure that PETAs understand both the principle and receptive experiences of any physical exam maneuvers they will be assessing."<br>Comments: Not universally applicable.	Mean (SD): 4.00 (1.35)<br>Results: Consensus achieved.	Reached consensus in Round 2.
3.4.6 In formative assessment, ensure consistent and accurate completion of an assessment instrument within individual SPs, and among groups of SPs performing the same task.	Mean (SD): 4.67 (.69)<br>Results: Consensus achieved.<br>"In formative assessment, ensure consistent and accurate completion of an assessment instrument within individual PETAs, and among groups of PETAs performing the same task."	Reached consensus in Round 1.	
3.4.7 In high stakes assessment, verify inter-rater reliability, in which a learner would achieve the same score	Mean (SD): 4.41 (1.06)<br>Comments: Consider "In high stakes assessment, verify inter-rater reliability, in which a	Mean (SD): 4.58 (1.17)<br>Comments: PETAS instruct, while SPs are more commonly used for high stakes	Mean (SD): 4.38 (1.19)<br>Results: Consensus not achieved due to Not Important/Applicable

when rated by different SPs.	learner would achieve the same score when rated by different PETAs."<br>Comments: Not universally applicable.	assessment. Inter-rater reliability may be important if assessing during instructional session.	selection.<br>Comments: No additional comments.
3.4.8 In high stakes assessment, verify intra-rater reliability, in which SPs would assign the same score to an identical performance at different points in time.	Mean (SD): 4.17 (1.29)<br>Comments: Not universally applicable. Consider: "In high stakes assessment, verify intra-rater reliability, in which PETAs would assign the same score to an identical performance at different points in time."	Mean (SD): 4.58 (1.17)<br>Comments: PETAS instruct, while SPs are more commonly used for high stakes assessment. Intra-rater reliability may be important if assessing during instructional session.	Mean (SD): 4.00 (1.29)<br>Results: Consensus not achieved due to Not Important/Applicable selection.<br>Comments: No additional comments.
**3.5 Reflection on the Training Process**			
3.5.1 Reflect on one's own training practices for future improvement (e.g., evaluation forms, debriefing, video review).	Mean (SD): 4.39 (.70)<br>Results: Consensus achieved.<br>Comments: Reflection supports improvement.	Reached consensus in Round 1.	

	Not Important/ Applicable (1)	Minimally Important (2)	Somewhat Important (3)	Very Important (4)	Critically Important (5)
**Domain 4: Program Management**					

	ROUND 1 RESULTS	ROUND 2 RESULTS	ROUND 3 RESULTS
**4.1 Purpose**			
4.1.1 Articulate a mission statement for the program.	Mean (SD): 4.33 (.84)<br>Results: Consensus achieved.<br>Comments: Mission statement should be clear to all stakeholders.	Reached consensus in Round 1.	
4.1.2 Develop program goals.	Mean (SD): 4.44 (.62)<br>Results: Consensus achieved.<br>"Develop program goals."	Reached consensus in Round 1.	
4.1.3 Identify measurable objectives for each goal (where applicable).	Mean (SD): 4.17 (.71)<br>Results: Consensus achieved.<br>"Identify measurable objectives for each goal (where applicable)."	Reached consensus in Round 1.	
**4.2 Expertise**			
4.2.1 Possess depth of knowledge in SP methodology.	Mean (SD): 4.67 (.49)<br>Results: Consensus achieved.<br>"Possess depth of knowledge in PETA methodology."	Reached consensus in Round 1.	
4.2.2 Advocate for the integration of SP methodology into the curriculum where	Mean (SD): 4.56 (.51)<br>Results: Consensus achieved.<br>"Advocate for the integration of	Reached consensus in Round 1.	

appropriate.	PETA methodology into the curriculum where appropriate."<br>Comments: Effective identification of resources is important to advocacy.		
4.2.3 Identify when SPs should be incorporated into a simulation activity.	Mean (SD): 4.00 (1.19)<br>"Identify when PETAs should be incorporated into an instructional session."<br>Comments: PETAs often instruct the assessment technique, while faculty separately provide context.	Mean (SD): 4.33 (.78)<br>Results: Consensus achieved.<br>Comments: No additional comments.	Reached consensus in Round 2.
4.2.4 Collaborate with subject matter experts to design SP cases, training, and assessment materials.	Mean (SD): 4.67 (.49)<br>Results: Consensus achieved.<br>"Collaborate with subject matter experts to design instructional sessions, training, and assessment materials."	Reached consensus in Round 1.	
4.2.5 Train SPs according to scenario or project parameters.	Mean (SD): 4.61 (.61)<br>Results: Consensus achieved.<br>"Train SPs according to instructional session or project parameters."	Reached consensus in Round 1.	
**4.3 Policies and Procedures**			
4.3.1 Develop and document policies to guide program activities.	Mean (SD): 4.44 (.62)<br>Results: Consensus achieved.<br>"Develop and document policies to guide program activities."	Reached consensus in Round 1.	
4.3.2 Develop and document	Mean (SD): 4.44 (.51)	Reached consensus in Round	

policies that take into consideration disability access and inclusion.	Results: Consensus achieved. "Develop and document policies that take into consideration disability access and inclusion."	1.	
4.3.3 Develop and document business processes and procedures, including but not limited to creating financial management, business, and strategic plans.	Mean (SD): 4.17 (.86)<br>Results: Consensus achieved. "Develop and document business processes and procedures, including but not limited to creating financial management, business, and strategic plans."	Reached consensus in Round 1.	
4.3.4 Ensure policies and procedures are kept current and accessible.	Mean (SD): 4.44 (.62)<br>Results: Consensus achieved. "Ensure policies and procedures are kept current and accessible."	Reached consensus in Round 1.	
4.3.5 Distribute policies and procedures to relevant stakeholders.	Mean (SD): 4.39 (.61)<br>Results: Consensus achieved. "Distribute policies and procedures to relevant stakeholders."	Reached consensus in Round 1.	
**New Additions**			
	4.3.6 "Develop and document policies and procedures to guide response if a learner identifies an unexpected finding."<br>Comments: Response when a learner detects a finding should be addressed in policy.	Mean (SD): 4.08 (.67)<br>Results: Consensus achieved. "Develop and document policies and procedures to guide responses to a learner-identified unexpected finding."	Reached consensus in Round 2.

	4.3.7 "Engage PETAs in developing and reviewing PETA program policies and procedures."<br>Comments: PETAs should have input in creation and review of PETA program policies and procedures.	Mean (SD): 3.67 (.89)<br>Comments: PETAs hold a distinct perspective in comparison to administration and/or SPEs.	Mean (SD): 3.85 (.69)<br>Comments: Consensus achieved.<br>Comments: No additional comments.
**4.4 Records Management**			
4.4.1 Collaborate with subject matter experts to develop a system for reporting learner performance to stakeholders (e.g., learners, curriculum developers, faculty, administration).	Mean (SD): 4.44 (.51)<br>Results: Consensus achieved.<br>Comments: Such collaborations enhance learning.	Reached consensus in Round 1.	
4.4.2 Ensure that policies are in place for case sharing and archiving.	Mean (SD): 4.28 (.75)<br>Results: Consensus achieved.<br>"Ensure that policies are in place for instructional material sharing and archiving."	Reached consensus in Round 1.	
4.4.3 Develop and document methods for securely storing, archiving, and destroying confidential data (e.g., SP records, learner data, video data, consent forms, release forms).	Mean (SD): 4.44 (.62)<br>Results: Consensus achieved.<br>"Develop and document methods for securely storing, archiving, and destroying confidential data (e.g., PETA records, learner data, video data, consent forms, release forms)."	Reached consensus in Round 1.	
**4.5 Team Management**			

4.5.1 Consult with legal, financial, and human resources experts to ensure that status of SPs (e.g., employee, independent contractor, volunteer) and compensation structure (if applicable) comply with institutional requirements.	Mean (SD): 4.72 (.46)<br>Results: Consensus achieved. "Consult with legal, financial, and human resources experts to ensure that status of PETAs (e.g., employee, independent contractor, volunteer) and compensation structure (if applicable) comply with institutional requirements."	Reached consensus in Round 1.	
4.5.2 Develop processes to identify, screen, interview, select, debrief, and maintain SPs and staff.	Mean (SD): 4.67 (.49)<br>Results: Consensus achieved. "Develop processes to identify, screen, interview, select, debrief, and maintain PETAs and staff."	Reached consensus in Round 1.	
4.5.3 Recruit and maintain a cohort of SPs that reflects the diversity of the people they represent in simulation activities.	Mean (SD): 4.56 (.62)<br>Results: Consensus achieved. "Recruit and maintain a cohort of PETAs that reflects the diversity of the people they represent in simulation activities."	Reached consensus in Round 1.	
4.5.4 Establish policies and procedures for the psychological, physical, and environmental safety of SPs, learners, staff, and faculty.	Mean (SD): 4.67 (.59)<br>Results: Consensus achieved. "Establish policies and procedures for the psychological, physical, and environmental safety of PETAs, learners, staff, and faculty."	Reached consensus in Round 1.	
4.5.5 Advocate for ongoing	Mean (SD): 4.44 (.70)	Reached consensus in Round	

professional development opportunities for all staff, including SPs.	Results: Consensus achieved. "Advocate for ongoing professional development opportunities for all staff, including PETAs." Professional development helps maintain and develop unique skill sets.	1.	
**4.6 Quality Management**			
4.6.1 Gather data regularly to assess the alignment of program activities with legislated, institutional, and program policies and procedures.	Mean (SD): 4.22 (.55)<br>Results: Consensus achieved. "Gather data regularly to assess the alignment of program activities with legislated, institutional, and program policies and procedures."	Reached consensus in Round 1.	
4.6.2 Gather feedback regularly from SPs, learners, faculty, and other users regarding the quality of services provided by the program.	Mean (SD): 4.39 (.50)<br>Results: Consensus achieved. "Gather feedback regularly from SPs, learners, faculty, and other users regarding the quality of services provided by the program."	Reached consensus in Round 1.	
4.6.3 Analyze data and other feedback in a timely manner.	Mean (SD): 4.28 (.57)<br>Results: Consensus achieved. "Analyze data and other feedback in a timely manner."	Reached consensus in Round 1.	
4.6.4 Implement changes for continuous improvement.	Mean (SD): 4.50 (.62)<br>Results: Consensus achieved. Comments: Quality improvement may address a	Reached consensus in Round 1.	

	process to review interactions, correct inaccuracies, and remove a PETA from a project as indicated.		
4.6.5 Inform stakeholders of changes made based on their feedback.	Mean (SD): 4.28 (.75)<br>Results: Consensus achieved.<br>"Inform stakeholders of changes made based on their feedback."	Reached consensus in Round 1.	
**New Additions**			
	4.6.6 "Provide the opportunity for PETAs to engage in self-evaluation."<br>Comments: PETAs should also reflect and engage in self-evaluation.	Mean (SD): 3.75 (.75)<br>Comments: No additional comments.	Mean (SD): 4.08 (.76)<br>Results: Consensus achieved.<br>Comments: No additional comments.

	Not Important/ Applicable (1)	Minimally Important (2)	Somewhat Important (3)	Very Important (4)	Critically Important (5)
**Domain 5: Professional Development**					

	ROUND 1 RESULTS	ROUND 2 RESULTS	ROUND 3 RESULTS
**5.1 Career Development**			
5.1.1 Develop and promote expertise in knowledge, skills, and attitudes related to SP-based simulation.	Mean (SD): 4.56 (.51)<br>Results: Consensus achieved.<br>Suggested Revision: "Develop and promote expertise in knowledge, skills, and attitudes related to PETA-based instructional sessions."	Reached consensus in Round 1.	
5.1.2 Develop and promote expertise in theories, principles, and processes of education and assessment relevant to the context of one's practice (e.g., medical education, nursing education, legal, and law enforcement training).	Mean (SD): 4.39 (.61)<br>Results: Consensus achieved.<br>"Develop and promote expertise in theories, principles, and processes of education and assessment relevant to the context of one's practice (e.g., medical education, nursing education, legal, and law enforcement training)."	Reached consensus in Round 1.	
5.1.3 Maintain membership in professional simulation societies (e.g., ASPE, ASPiH, INACSL, SESAM, SSH).	Mean (SD): 4.17 (.79)<br>Results: Consensus achieved.<br>"Maintain membership in professional simulation	Reached consensus in Round 1.	

	societies (e.g., ASPE, ASPiH, INACSL, SESAM, SSH) or medical education societies.”		
5.1.4 Engage in educational opportunities (e.g., professional conferences, courses, degree programs, certifications).	Mean (SD): 4.44 (.51)<br>Results: Consensus achieved.<br>“Engage in educational opportunities (e.g., professional conferences, courses, degree programs, certifications).”	Reached consensus in Round 1.	
5.1.5 Develop personal management skills (e.g., time management, wellness strategies, career planning).	Mean (SD): 4.11 (.83)<br>Results: Consensus achieved.<br>“Develop personal management skills (e.g., time management, wellness strategies, career planning).”	Reached consensus in Round 1.	
5.1.6 Seek out opportunities for career mentoring.	Mean (SD): 4.06 (.77)<br>Results: Consensus achieved.<br>“Seek out opportunities for career mentoring.”	Reached consensus in Round 1.	
**5.2 Scholarship**			
5.2.1 Develop an understanding of the range of opportunities for scholarship in SP methodology.	Mean (SD): 3.94 (.87)<br>Results: Consensus achieved.<br>“Develop an understanding of the range of opportunities for scholarship in PETA methodology.”	Reached consensus in Round 1.	
5.2.2 Identify and/or develop new contexts for SP methodology.	Mean (SD): 4.00 (.84)<br>Results: Consensus achieved.<br>“Identify and/or develop new contexts for PETA	Reached consensus in Round 1.	

	methodology."		
5.2.3 Contribute to the evolution of best practices through innovation, research, and dissemination of emerging methods in various venues (e.g., publications, presentations).	Mean (SD): 4.39 (.85)<br>Results: Consensus achieved.<br>"Contribute to the evolution of best practices through innovation, research, and dissemination of emerging methods in various venues (e.g., publications, presentations)."	Reached consensus in Round 1.	
**5.3 Leadership**			
5.3.1 Promote understanding and development of SP methodology locally, nationally, and internationally.	Mean (SD): 4.17 (.79)<br>Results: Consensus achieved.<br>"Promote understanding and development of PETA methodology locally, nationally, and internationally."	Reached consensus in Round 1.	
5.3.2 Mentor and support SPs and other SP educators within one's institution and within the community of practice.	Mean (SD): 4.39 (.78)<br>Results: Consensus achieved.<br>"Mentor and support PETAs and other PETA educators within one's institution and within the community of practice."	Reached consensus in Round 1.	
5.3.3 Seek out and advocate for growth of leadership skills (e.g., collaboration, team building, change management, interpersonal effectiveness, conflict resolution).	Mean (SD): 4.33 (.84)<br>Results: Consensus achieved.<br>"Seek out and advocate for growth of leadership skills (e.g., collaboration, team building, change management, interpersonal effectiveness, conflict resolution)."	Reached consensus in Round 1.	
